# Longitudinal decline of leukocyte telomere length in old age and the association with sex and genetic risk

**DOI:** 10.18632/aging.100995

**Published:** 2016-07-07

**Authors:** Kari Berglund, Chandra A. Reynolds, Alexander Ploner, Lotte Gerritsen, Iiris Hovatta, Nancy L. Pedersen, Sara Hägg

**Affiliations:** ^1^ Department of Medical Epidemiology and Biostatistics, Karolinska Institutet, 171 77 Stockholm, Sweden; ^2^ Department of Psychology, University of California-Riverside, Riverside, CA 92521, USA; ^3^ Department of Clinical Psychology, Faculty of Social Sciences, Utrecht University, Utrecht, the Netherlands; ^4^ Department of Biosciences, Viikki Biocenter, University of Helsinki, Helsinki, Finland; ^5^ Department of Health, National Institute for Health and Welfare, Helsinki, Finland

**Keywords:** telomere length, longitudinal decline, aging, genetic risk score, latent growth curve

## Abstract

Telomeres are DNA-protein structures at the ends of chromosomes. Leukocyte telomere length (LTL) shortening has been associated with advanced age. However, most studies use cross-sectional data, hence, the aim of our study was to model longitudinal trajectories of LTL attrition across 20 years at old age. Assessments of LTL were done by qPCR in SATSA (Swedish Adoption/Twin Study of Aging; N=636 individuals). Cross-sectional and longitudinal associations with age were estimated, the latter using latent growth curve analysis. A genetic risk score (GRS) for LTL was further assessed and included in the models. We confirmed an inverse cross-sectional association of LTL with age (B=−0.0022 T/S-ratio; 95% CI: −0.0035, −0.0009, p-value=0.0008). Longitudinal LTL analyses adjusted for sex (1598 samples; ≤5 measurements) suggested modest average decline until 69 years of age but accelerating decline after 69 years, with significant inter-individual variation. Women had on average ∼6% T/S-ratio units longer LTL at baseline, and inclusion of the GRS improved the model where four risk alleles was equivalent to the effect size difference between the sexes. In this cohort of old individuals, baseline LTL varied with age, sex and genetic background. The rate of change of LTL accelerated with age and varied considerably between individuals.

## INTRODUCTION

Telomeres are DNA-protein structures of tandem hexanucleotide repeats at the ends of eukaryotic chromosomes, providing protection from degradation and recombination during cell division [1]. In somatic cells, telomeres are inadequately replicated, and, as a result, telomere length declines with each cell division [2]. Leukocyte telomere length (LTL) in humans is inversely associated with age and is influenced by genetic [3] and environmental factors [4]. Moreover, telomeres are shorter in men than in women; a consequence of an accelerated attrition rate in men throughout the life course [5-9]. Shorter telomeres have been implicated in several diseases, including cardiovascular disease, but it is unclear what direct impact, if any, telomeres have on the development of age-related diseases [8, 10-14].

A recent meta-analysis of 124 cross-sectional studies has shown that telomere lengths are significantly shorter with age [15], and at least six longitudinal studies report a decrease in telomere length with advancing age [16-21]. However, previous efforts have had shorter follow-up time and only included two to three time points per study subject, which is insufficient for estimating trajectories of change. In the present study, we examine both the cross-sectional and longitudinal associations between LTL and chronological age in elderly Swedish twins with up to five LTL measurements per individual across 20 years. Thus, we sought to characterize LTL trajectories with advancing age, and to test whether individual differences in trajectories may be accounted for in part by sex and a genetic risk score (GRS) for LTL identified in a prior genome-wide association study [3].

## RESULTS

### Cross-sectional analysis

The Swedish Adoption/Twin Study of Aging (SATSA) [22] is a longitudinal study started in 1984 including twins from mid-life and onwards. It consists of twin pairs reared apart matched with twin pairs reared together, and is collected from all over Sweden. The individuals are followed up to 30 years with in-person testing (IPT) of cognitive ability, physical fitness, anthropometrics, health data and questionnaires up to 10 times. Data on LTL are available from IPT 3, 5, 6, 8, and 9, and were attained by qPCR techniques (see Methods for more details). Basic characteristics of a cross-sectional sample of LTL measures at baseline (n=636 individuals from same sex twin pairs with 84 monozygotic and 152 dizygotic complete pairs) are presented in Table 1. Through linear regression adjusted for family relatedness and sex, an inverse association between LTL and age was found (B=−0.0022 T/S-ratio/year; 95% confidence interval [CI]: −0.0035, −0.0009, p-value=0.0008) and female sex was associated with a greater overall LTL (0.0499; 95% CI: 0.0200, 0.0797; p-value=0.001). Stratifications on zygosity did not significantly impact the model (data not shown). By using genotype data, an un-weighted GRS summarizing risk alleles from seven single nucleotide polymorphisms (SNPs) associated with LTL [3] was calculated per individual (*M*=8.42, *SD*=1.43; Supplementary Table 1).

**Table 1:** SATSA cross-sectional characteristics

	SATSA (N = 636)
Years of data collection	1992 - 2012
Telomere length (mean ± sd)^$^	0.70 ± 0.17
Age (mean ± sd)	68.86 ± 9.67
Women (%)	372 (58%)
GRS (mean ± sd)*	8.42 ± 1.43

$ Mean telomere length is adjusted for batch effect and re-scaled back to T/S-ratio.

* Genetic information was available for 585 individuals. GRS: genetic risk score.

The overall effect of the GRS on LTL in SATSA (−0.046 SD-decrease in T/S-ratio per additional risk allele; 95% CI: −0.111, 0.019) was similar to the effect seen in the original LTL genome-wide association study (GWAS) (−0.070; 95% CI: −0.077, −0.063) although the CI's were wider (Supplementary Figure 1, upper panel). Hence, the GRS did not significantly contribute to the cross-sectional model and did not alter the estimates markedly (Supplementary Table 2). Additional sensitivity analyses are presented in Supplementary Tables 2 and 3.

### Longitudinal analysis

Longitudinal sample characteristics are reported by measurement occasions and sex in Table 2. Women in general have a higher mean LTL than men at similar ages, whether by measurement occasions or by IPT's (Supplementary Table 4). The distribution of longitu-dinal samples across the 636 individuals was 31.1% (N=198) with one measurement; 21.4% (N=136) with two; 21.7% (N=138) with three; 16.7% (N=106) with four; and 9.1% (N=58) with five measurements, hence, 47% had at least three measurements available.

**Table 2:** SATSA longitudinal characteristics by sex and measurement occasions

	Measurement Occasion				
	1^st^ Measure	2^nd^ Measure	3^rd^ Measure	4^th^ Measure	5^th^ Measure
Men	(N=264)	(N=181)	(N=128)	(N=66)	(N=22)
Telomere length (mean±sd)^$^	0.68±0.18	0.70±0.12	0.70±0.15	0.68±0.09	0.70±0.19
Age (mean±sd)	67.44±8.79	71.84±8.70	74.36±7.93	76.52±7.82	78.42±5.74
Women	(N=372)	(N=257)	(N=174)	(N=98)	(N=36)
Telomere length (mean±sd)^$^	0.72±0.16	0.72±0.13	0.72±0.13	0.73±0.13	0.68±0.16
Age (mean±sd)	69.86±10.13	73.75±9.45	76.55±8.85	78.73±8.52	80.91±8.07

$ Mean telomere length is adjusted for batch effect and re-scaled back to T/S-ratio.

**Figure 1:**
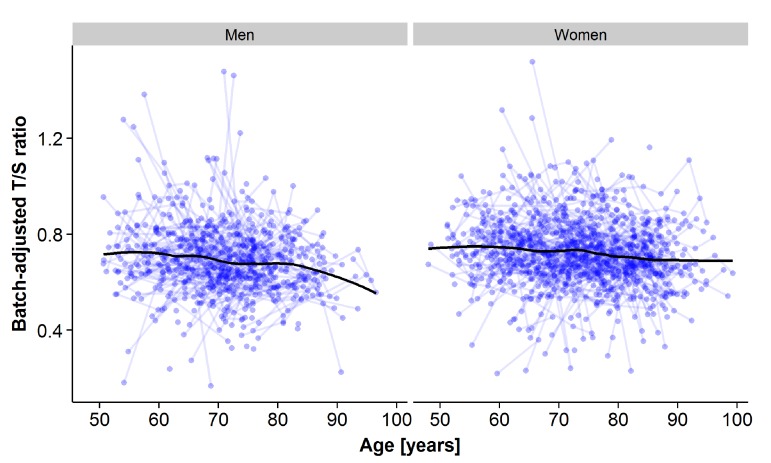
Plots of all leukocyte telomere length (LTL) samples across measurement points for the longitudinal analyses for men (left panel) and for women (right panel). The x-axis represents age at sample testing, and the y-axis represents the plate-adjusted residuals of LTL re-scaled back to T/S-ratio. A loess smoothing line calculated from a weighted regression over age for all samples is shown.

An inverse relationship between LTL and age was seen when plotting individual trajectories of all samples stratified by sex (Figure 1). The overall trend, presented here as a smoothing curve, is close to linear for both men and women, with similar slopes, but with a slightly lower intercept and accelerating attrition in older age in men. Individual trajectories with linear trends, including a split at the centering age defined as the median age at IPT3 (69.3 years), are displayed in the supplement (Supplementary Figure 2).

**Figure 2:**
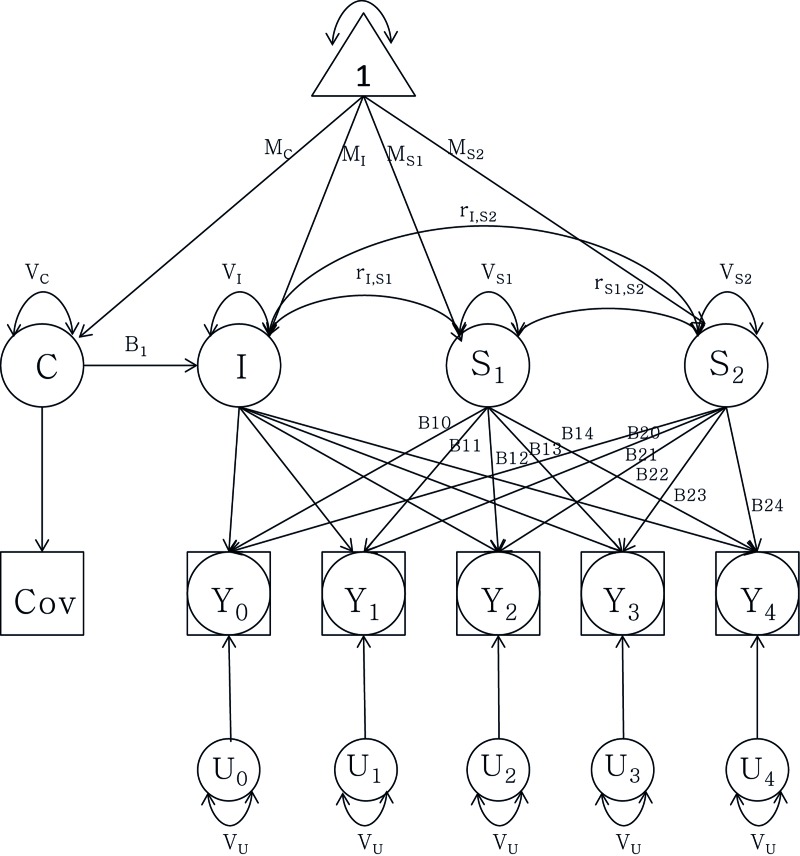
Latent growth curve analysis of telomere length attrition with a group intercept (I) and two slopes (S1) and (S2) as well as sex as covariate (C). Observed data are denoted by Y0 through Y4. MI: mean intercept; MS1: mean slope 1; MS2: mean slope 2; MC: mean covariate. V stands for variance of each component respectively. r denotes the correlation between the intercept and the slopes. B1t through B2t represent the age basis coefficients with change over time t. U0 through U4 represent random components from the telomere length measurements, constrained to be equal for each assessment.

To quantify telomere attrition longitudinally, latent growth curve (LGC) models were tested (Figure 2, Methods, and Supplementary Data). In brief, an LGC model is a mixed model including three variables: 1) the intercept with the LTL value at the age of 69.3 years, 2) a linear slope before the centering age (69.3 years), and 3) a linear slope after the centering age. Variances and cross-correlations were presented for all three variables and twin relatedness were adjusted for by estimating between- and within-pair variances. At first, an intercept-only (no change) model (Model 1) was compared to a one-slope model without any change point included (Model 2), allowing for a linear rate of change across the whole age span. The improvement in fit was significant (p-value=2·10-8; Table 3). Model 2 also revealed significant variation between twin pairs in LTL change, suggestive of familial factors (Supplementary Table 5). Next, the one-slope model (Model 2) was compared to a two-slope model allowing for two linear rates, one before and one after age 69.3, respectively (Model 3). Again, the fit was substantially improved (p-value=0.006; Table 3). The fixed effect (average) for slope 1 in model 3 (young-old age<69.3) was not significant (p-value=0.30), but it was for slope 2 (old-old age>69.3; B=−0.0021 T/S-ratio/year; 95% CI: −0.0034, −0.0008, p-value=0.002). The variance around the intercept for both familial (between twin pairs) and environmental (within twin pair) effects contributed strongly (p-value<0.001 for both effects) to the model (Table 4). However, there was also variation detected for both slope 1 and 2 for the within-pair random effect (p-value<0.01 and <0.05 respectively), suggesting that non-familial/environmental factors are important to rate of change at both young- and old-old age, although the effect was bigger at young-old age. Indeed, sensitivity analyses indicated that it was necessary to retain the sets of between and within-pair random effects for both slope 1 and slope 2 (Δχ^2^ p-value<0.0005), indicating individual differences in change across both age periods. Altogether, the average difference between slope 1 and 2 in model 3 was not great, as was obvious from the raw trajectories depicted (Figure 1 and Supplementary Figure 2). Rather, the variation around the slopes was appreciable and larger for slope 1 than slope 2 (see Supplementary Table 6 for complete model estimates). Further sensitivity analyses suggested that the same trends remained when altering the centering age to be five years earlier or later, and again, as in the cross-sectional analysis, zygozity did not matter (data not shown). Finally, women had on average 0.0409 higher T/S-ratio than men (p-value=0.0005; Table 4), corresponding to a 6% difference in baseline T/S-ratio at 69.3 years. The estimate was similar although smaller to the cross-sectional estimate (0.0499). However, no interaction was seen between sex and the rate of decline in old-old age (Table 5).

**Table 3:** Latent growth curve models for leukocyte telomere length and age (N=1598)

Models tested						
Model	−2LL	Parms	AIC	BIC	Δ-2LL	*df*
1: Intercept only	−1865.9	5	−1855.9	−1836.2	--	--
2: Intercept + slope 1	−1910.5	10	−1892.5	−1857.1	44.6^§^	5***
3: Intercept + slope 1 & 2	−1930.4	17	−1900.4	−1841.4	19.9^£^	7**

§ Model 1 vs. model 2.

£ Model 2 vs. model 3.

Chi-square tests for deviance: **p-value<0.01; ***p-value<0.001. In all models, sex as covariate as well as random effects between twin pairs and between individuals were included. LL: log-likelihood. Parms: parameters; AIC: Akaike Information Criterion; BIC: Bayesian Information Criterion; *df*: degrees of freedom.

**Table 4:** Fixed and random effects of the full spline two-slope model (Model 3) from latent growth curve analysis of leukocyte telomere length and age

Fixed Effects			
Intercept (estimate (95% CI))	Slope 1 (estimate (95% CI))	Slope 2 (estimate (95% CI))	Sex (estimate (95% CI))
0.7287 (0.7095-0.7479)***	−0.0012 (−0.0033-0.0010)	−0.0021 (−0.0034- −0.0008)**	−0.0409 (−0.0637- −0.0182)***

Random Effects					
varI_t_	varI_p_	varS_1t_	varS_1p_	varS_2t_	varS_2p_
0.0060***	0.0082***	0.00006**	0.00001	0.00002*	0.00002

* p-value<0.05;

** p-value<0.01;

*** p-value<0.001;

Note. Random effects: varI = variance around the intercept; varS_1_ = variance around slope 1; varS_2_ = variance around slope 2; In all models, sex as covariate as well as random effects between twin pairs (subscript ‘p’) and within twin pairs (subscript ‘t’) were included.

**Table 5:** Additional adjusted two-slope models (Model 3) of leukocyte telomere length and age (N=1504)

	Models tested						
Model	−2LL	Parms	AIC	BIC	Reference model	Δ-2LL	*df*
3	−1834.2	17	−1802.2	−1740.1	--	--	--
3+slope2*sex	−1834.4	18	−1800.4	−1734.4	3	0.2	1
3+GRS	−1843.1	18	−1809.1	−1743.2	3	8.9	1**
3+GRS+slope2*GRS	−1843.9	19	−1807.9	−1738.0	3+GRS	0.8	1
3+GRS+sex*GRS	−1843.2	19	−1807.2	−1737.3	3+GRS	0.1	1

Chi-square tests for deviance: **p-value<0.01; In all models, sex as covariate as well as random effects between twin pairs and between individuals were included. LL: log-likelihood. Parms: parameters; AIC: Akaike Information Criterion; BIC: Bayesian Information Criterion; *df*: degrees of freedom; GRS: Genetic Risk Score.

### Genetic risk scores in longitudinal models

**Figure 3:**
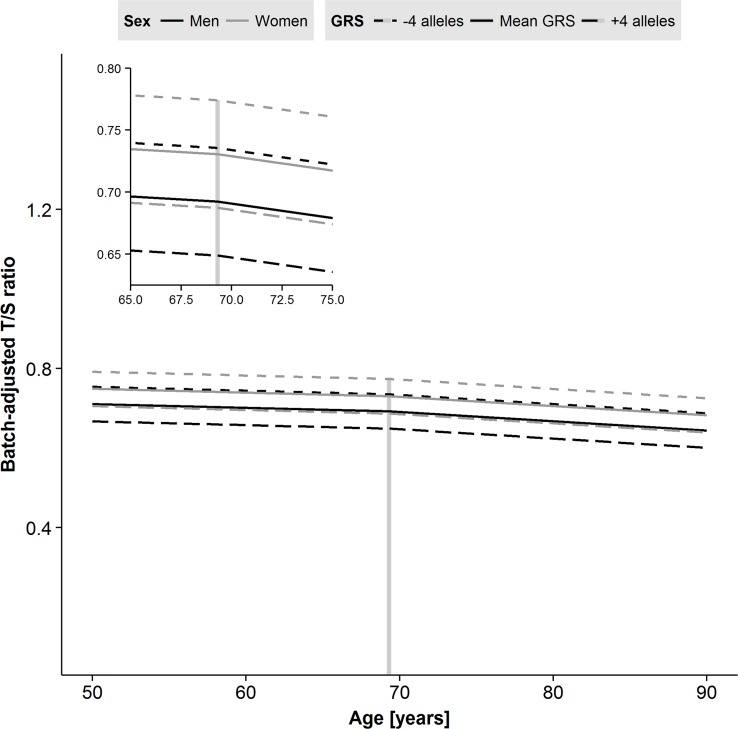
Predicted trajectories for men and women based on parameter estimates from the two-slope model of leukocyte telomere length (LTL) including sex and genetic risk score (GRS) effects. Male sex and addition of risk alleles in the GRS each result in shorter LTL. A decline after the centering age of 69.3 years is apparent for both men and women. Age in years is on the x-axis, and the plate-adjusted residuals of LTL re-scaled back to T/S-ratio is on the y-axis. The dashed line indicates centering age and the left corner panel is a zoomed version.

To test whether genetic predisposition towards shorter LTL had an impact in the longitudinal model, the GRS for LTL was entered into the fixed effects of the two-slope model (Supplementary Table 7). The fit improved markedly (p-value=0.003), but GRS did not interact with sex or age (Table 5). The different trajectories seen for model 3 depending on age, sex and GRS parameter estimates are displayed in Figure 3. Men have generally shorter LTL than women, and the trajectories are parallel. One risk allele increase of the GRS results in additional −0.011 decrease in T/S-ratio (95% CI: −0.018, −0.004, p-value=0.003), and four additional risk alleles are equivalent to the difference in T/S-ratio in men compared to in women. Overall, the GRS estimate from the LGC model is similar to the estimate from the cross-sectional model; however, the CI's are smaller (Supplementary Figure 1, lower panel). We further investigated effects of individual genetic variants (those included in the GRS) on the two-slope model and found the *TERC* locus to be the most important contributor with significant effect on its own (B=−0.020; 95% CI: −0.037, −0.003, p-value=0.02, Supplementary Figure 1, lower panel). However, none of the SNPs was individually as good as the GRS was for improving the

### Telomere elongation

**Figure 4:**
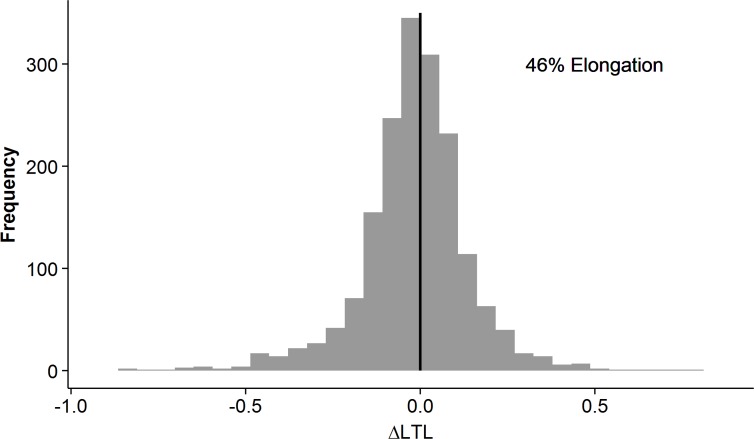
Individual relative leukocyte telomere length (LTL) change in the longitudinal cohort. The difference in LTL measurement between any two time-points in the same individual is on the x-axis. The frequency is on the y-axis. Telomere elongation is exhibited in samples with delta LTL>0, and telomere attrition is exhibited in samples with delta LTL<0. The sample distribution shows an overall elongation in 46%.

Longitudinally, many individuals exhibited telomere elongation from one occasion to the next (Figure 4). Elongation was seen in 46% of the within-individual sample comparisons, including all possible combinations, ranging from 44-47% depending on the number of years between measurements (Supplementary Figure 3). The coefficient of variation from the qPCR analyses was ∼7%, suggesting that the elongation seen was likely a biological phenomenon although technical bias from measurement imprecision and/or possible differences in sample collection between IPT's could not be completely ruled out.

## DISCUSSION

In the current study, we first examined the cross-sectional associations between LTL and age, and, like previous reports, we found an inverse relationship with increasing age. Second, using LGC analysis with up to five measurements across 20 years, we found that LTL decreases with age in a two-slope model with a small acceleration of decline after 69.3 years of age. Men have shorter telomere lengths than women, and genetic variation has an additional influence on overall LTL.

Several earlier studies have reported an inverse association between age and telomere length [15-20, 23], as did we, and we further demonstrated that women have longer LTL, which is in line with earlier research [5-7, 15, 23]. Taking our results and prior literature together, shorter telomeres in men could result from very small but consistent attrition throughout adulthood rather than a steeper decline compared to women in old age. Moreover, previous literature from cross-sectional and longitudinal studies has suggested a linear relationship between telomere length and age [15-20]. We found both the one-slope and the two-slope models to be significant, with a substantially better fit of the latter. While the overall average trend was linear, there was systematic variability around the average trend, better described in a two-slope model accounting for more individual differences.

The magnitude of this age-related decline was small overall, and with slight acceleration in the old-old. This observation is in line with earlier research in the field where faster decline in LTL is believed to take place in childhood and old age [23]. The age-related telomere loss in SATSA was similar in both cross-sectional and longitudinal analyses, and somewhat smaller than earlier longitudinal estimates of T/S-ratio attrition rate [19]. A likely reason for differing results may be measurement imprecision from using the qPCR technique and fluctuations in lymphocyte sub-populations. The two-slope trajectory analyses supported both familial and non-familial influences on LTL, with equal contributions to average LTL level (at age 69) and non-familial sources featuring more prominently in the change before age 69 than after age 69. This suggests that in young-old age, individual-specific lifestyle factors may prove more relevant to accelerated LTL shortening above and beyond familial and environmental contributions to overall LTL; however, in old-old age, familial factors may become increasingly salient to accelerated LTL shortening. Moreover, we note that the variation in rate of change was larger in young-old age; hence, evaluating variation in trajectories beyond the assumption of simple linearity and average trends is important for understanding etiological underpinnings.

Previous studies have investigated the heritability of LTL; a meta-analysis of five European ancestry cohorts reported it to be 0.70 [24], our study contributes new information on how telomere attrition *rates* may vary between twins. The same meta-analysis also looked at LTL correlations in spouses and found it to be substantial with larger effects in old age, in line with our findings of environmental contributions to the slopes.

Moreover, the GRS significantly improved the two-slope model when added to the fixed effect, suggesting that genetic background contributes to the overall LTL, but not its rate of change: we did not observe any significant interaction between slope 2 and GRS. In addition, when testing the individual gene effects on the LTL trajectories, we found them to be proportional to the genetic variant effect sizes, with the *TERC* gene (telomerase RNA component) as the most important contributor to the model. The estimated decreased effect in T/S-ratio from one additional risk allele increase in GRS was similar in both longitudinal (−0.011) and cross-sectional models (−0.015). Furthermore, we could not find any interaction between sex and GRS, which indicates that the genetic effect on LTL attrition is equal in both men and women. However, the genetic risk component can have larger effects than sex on telomere shortening; the addition of four risk alleles cancel out the sex effect indicating that individual predisposition to LTL attrition is of great importance.

### Telomere elongation

Telomere elongation has been observed and discussed in other longitudinal cohorts with LTL assayed by qPCR technique [25-29]. They all report evidence of small increases in telomere length, consistent with the lengthening that we observed. It still remains unclear what causes leukocyte telomere elongation. However, a recent mouse study demonstrated that, although increased stress levels accelerate telomere attrition, telomeres recover back to normal length when the stress subsides [30]. Nonetheless, the interesting question is whether the individual cells extend their telomeres by up-regulating the telomerase enzyme or whether there is renewal of the leukocyte population from the stem cell pool, leading to overall longer LTL due to faster turnover and larger number of young cells. In our sample, the elongation exceeds what would be expected from the coefficient of variation (6.98%) of the qPCR method, but technical variability could also be inferred from differences in sample collection over time and changes in leukocyte turnover. Additional research is necessary to explore why telomere elongation has been observed in this and in other studies.

### Strengths and limitations

The strength of this study is the inclusion of repeated LTL samples (up to five time points) over a 20-year time period, allowing exploration of trajectories as opposed to the maximum of three time points in previous studies [17, 18]. Moreover, the study design with twin pairs allowed us to investigate the familial and non-familial contributions to individual differences; at the same time, inclusion of GRS enabled quantification of genetic propensity to overall telomere shortening.

Limitations of the study include the generalizability of LTL to other tissues. Although LTL is strongly correlated with telomere length in muscle, skin, and subcutaneous fat, it is unclear what sort of longitudinal decline telomeres may experience in other cell types that undergo mitosis at different frequencies [31]. Additionally, because of our focus on older Swedish adults, we cannot generalize our results to younger adults or other ancestries. Finally, our slope estimates in both the cross-sectional and longitudinal models are based on relative attrition measures due to use of the qPCR-based LTL measurement. This limits the comparability of our results with other studies using other measurement techniques.

In summary, we present for the first time trajectories of longitudinal telomere decline in old age using a twin design including investigation of genetic contributions. The annual decline is small and linear with slightly accelerated decline after 69 years. Male sex and inclusion of a GRS for LTL in the models were independently associated with shorter telomeres.

## METHODS

### Study samples

The Swedish Twin Registry (STR) [32] is a population-based national registry established in the late 1950s and consists of twins born 1886-2000. SATSA (The Swedish Adoption/Twin Study of Aging) is a sub-study of STR and was started in 1984 with a first questionnaire sent out to twins reared apart matched with twins reared together (N=2018) [22]. Two years later the first in-person testing (IPT) was made on 645 individuals (303 pairs) over the age of 50 years. Following approximately every third year, a new IPT was administered and a total of ten IPTs were done until 2014 with questionnaires, anthropometric measures, blood sampling, physical function measurements and cognitive testing. More information on the SATSA study, including a list of references, can be found online (http://ki.se/en/meb/satsa-the-swedish-adoptiontwin-study-of-aging). LTL assessments were done in individuals attending at least one of IPT 3, 5, 6, 8 or 9 (N=636 individuals corresponding to 1598 samples). Cross-sectional LTL data was based on the first available measure. Genotype information was gathered through the Illumina CardioMetabochip [33] and was available for 585 individuals.

### Telomere assessment

LTL measurements were derived from blood leukocytes using a qPCR assay, comparing a telomere length PCR product (T) against a PCR product of a reference gene (S) to produce a T/S-ratio [34]. A detailed description of the LTL assessment is presented in the Supplementary Data.

### Genetic risk score calculation

An individual, non-weighted GRS was calculated using seven genetic variants previously known to be associated with LTL [3]; directly genotyped (rs2736100, rs2281929) and imputed variants (rs11125529, rs10936599, rs7675998, rs9420907, rs8105767) (Supplementary Table 1). The SATSA study was not included in the original GWAS. For each individual, all risk alleles for shorter LTL were counted and summed. The GRS variable was centered on its mean before inclusion into the models.

### Statistical analyses

In the telomere samples, plate ID was used to adjust for batch effect and residuals were re-scaled back to T/S-ratio by adding the mean to each value (Supplementary Data, Supplementary Figure 4). Hence, the weighted estimates from the models are reported as B values not to be confused with standardized β values. Moreover, samples were excluded if the T/S-ratio exceeded four SD's from the mean within the study (cross-sectional: [N=6]) or within a given age group (longitudinal: 50-59 years [N=1], 60-69 years [N=3], 70-79 years [N=4], 80+ years [N=3]).

Linear regressions with re-scaled LTL residuals as the outcome variable and sex as a covariate were performed on the cross-sectional data to evaluate age and LTL relationships. Twinness was controlled for by robust sandwich estimates of standard errors using *PROC SURVEYREG* including cluster on twin pair in SAS 9.4 (SAS Inc, Cary, NC).

Latent growth curve models have been used previously with SATSA data to model age changes in cognitive functioning [35, 36]. Specifically, regression models were fitted to each individual's longitudinal LTL trajectory, resulting in an average model for the sample (fixed effects) plus individual deviations from the average model (random effects). Familial influences on LTL were accounted for by allowing for random effects between twin pairs and non-familial/environmental factors estimated via within-pair random effects. Sex was included in all models. A best-fit model was selected via chi-square difference tests (Δχ^2^), comparing the −2 log-likelihood (−2LL) of an intercept-only model, a one-slope model, and a two-slope model, with age variables centered at the median age at IPT3 (69.3 years). The one-slope model estimated a single linear rate of change effect across age, while the two-slope model allowed for two linear rates, before and after age 69.3, respectively. Comparisons of additional two-slope models with GRS were carried out likewise. Individual trajectories were plotted together with a smoothing curve defined by a locally weighted regression model, summarizing the average behavior of LTL as a function of age without explicit assumptions about its overall shape. Predicted trajectory curves were calculated and further plotted from the parameter estimates of the two-slope model. All LGC analyses were performed using *PROC MIXED* in SAS 9.4, and R version 3.2 was used to create figures. The cut-off level used for overall significance were p<0.05 as is commonly used for these types of models [37].

## SUPPLEMENTAL DATA FIGURES AND TABLES


