# Supplementary material for: Longitudinal decline of leukocyte telomere length in old age and the association with sex and genetic risk
Source: Aging (Albany NY). 2016 Jul 7;8(7):1398–407. doi: 10.18632/aging.100995 (PMC4993338; doi:10.18632/aging.100995)
Supplement: Supplementary file 1 [file aging-08-1398-s001.pdf]

SUPPLEMENTARY DATA

Leukocyte telomere length measurement…………….
Batch effect adjustments of leukocyte telomere length..
Additional cross-sectional models tested……………...
Latent Growth Curve (LGC) Analysis…………………
References……………………………………………...
Supplementary Tables………………………………….
Supplementary Figures…………………………………
Appendix……………………………………………….

Leukocyte telomere length measurement

Leukocyte telomere length (LTL) was measured from DNA extracted from peripheral blood. We used a qPCR-based method [1], as described previously [2-4]. We used β-hemoglobin (Hgb) as a single copy reference gene. Separate reactions for telomere and Hgb reaction were carried out in paired 384-well plates in which matched sample well positions were used. Ten nanograms of DNA was used for each reaction, performed in triplicate. Every plate included a 7-point standard curve, which was used to create a standard curve and to perform absolute quantification of each sample. Samples and standard dilutions were transferred into the plates using a multichannel pipet and dried overnight at room temperature. Specific reaction mix for telomere reaction included 270 nM tel1b primer (5´-CGGTTT(GTTTGG)5GTT-3´) and 900 nM tel2b primer (5´-GGCTTG(CCTTAC)5CCT-3´), 0.2X SYBR Green I (Invitrogen), 5 mM DTT (Sigma-Aldrich), 1% DMSO (Sigma-Aldrich), 0.2 mM of each dNTP (Fermentas), and 1.25 U AmpliTaq Gold DNA polymerase (Applied Biosystems) in a total volume of 15 µl AmpliTaq Gold Buffer II supplemented with 1.5 mM $MgCl_2$. Hgb reaction mix included 300 nM Hgb1 primer (5´-GCTTCTGACACAACTGTGTTCACTAGC-3´) and Hgb2 primer (5´-CACCAACTTCATCCACGT TCACC-3´) in a total volume of 15 µl of iQ SyBrGreen supermix (BioRad). The cycling conditions for telomere amplification were: 10 minutes at 95 °C followed by 25 cycles at 95 °C for 15 s and 54°C for 2 min with signal acquisition. The cycling conditions for Hgb amplification were: 95 °C for 10 min followed by 35 cycles at 95 °C for 15 s, 58 °C for 20 s, 72 °C for 20 s with signal acquisition. Reactions were performed with CFX384 Real-Time PCR Detection System (Bio-Rad). Melt-curve analysis was carried out in the end of the run to ensure specific primer binding.

We used the Bio-Rad CFX Manager software to perform quality control, and samples with standard deviation of >0.5 between triplicates were omitted from the analysis. Plate effect was taken into account by analyzing five genomic DNA control samples on every plate. We normalized the telomere and Hgb signal values separately to the mean of these control samples before taking the T/S ratio. A few samples failed in the qPCR assay (N=48, ~3%) and were thus excluded from further analysis.

Batch effect adjustments of leukocyte telomere length

LTL measurements by qPCR techniques are sensitive to differences in the analyses protocols. In our samples, DNA extraction was done in one lab with one protocol, and the LTL assessments were then made in another lab using the protocol described above. Nevertheless, the T/S ratio reported may vary depending on which plate a given sample was run on, known as a batch effect. To explore a potential batch effect, linear regressions with LTL T/S-ratio as dependent variable and age, sex and dummy variables for the different plate IDs as independent variables revealed two plates with significant technical variation. A box plot divided by the different plate IDs were made to illustrate the batch effect (Supplementary Figure 4A). Thus, the T/S-ratio measurements were adjusted for plate number (two plates out of 22 had significant variation) by calculating residuals from the linear regression of T/S-ratio on plate ID (Supplementary Figure 4B), and residuals were then re-scaled back to T/S-ratio by adding the global mean to each value.

Additional cross-sectional models tested

In all additional models tested, LTL decreased with advancing age (Supplementary Table 2). Model 1, the crude model, and Model 3, including sex and GRS, showed effect estimates similar to the main model with sex as a covariate, presented in the main text. However, Model 2, which also included an interaction term between sex and age, did not fit well. Overall, sex contributes to the model but the GRS does not, nor are there significant interactions.

In sex-stratified models, LTL decreased with advancing age for both men and women (p-value ≤0.02 for both models). Men experienced a slightly accelerated decline in LTL; however, the differences in yearly telomere attrition rates between men and women were not significant (Supplementary Table 3).

Latent Growth Curve (LGC) Analysis

All LGC analyses were performed using PROC MIXED in SAS 9.4, and the SAS code is provided as an appendix at the end of the Supplementary Data. First, the centering age was selected to be 69.3 years because

it was the median age at baseline (IPT3). (This was later tested using sensitivity analyses of the models with centering ages +/- 5 years from 69.3 year as well as with exclusion of IPT3 measures.) Next, the data was coded so that all samples contributing to slope 1 (age < 69.3 years) were marked and likewise for all samples contributing to slope 2 (age > 69.3 years). An intercept only model was run were all samples at baseline contributed, even the individuals with only one time-point available contributed to this model. Then a simple linear slope was added to the model which improved model fit as described in the main text. Here all samples with more than one time point contributed to the slope. Finally a two-slope model was tested which had an even better fit to the data. To contribute to both slopes, an individual had to have samples from both before and after the centering age (69.3 years) and also at least three time points, many had up to five time points available. An individual with only two time points contributed to one of the slopes only. For each new model run, the improved model fit was confirmed using log-likelihood ratio test with comparisons to the prior model. All models included sex as covariate and adjustments for twinness by inclusion of random effects for within- and between twin pairs. When the genetic for within- and between twin pairs. When the genetic risk score was added to the model it was further improved, but only for level of TL at age 69.3, which meant that the contribution was on the intercept only and not on change. Because of lack of samples with genetic markers was available, the genetic model was run and tested on a smaller set of individuals and comparisons had to be made to the same smaller set of individuals in the prior comparison model(s) as well**.**

RERERENCES

1. Cawthon RM. Telomere measurement by quantitative PCR. Nucleic Acids Res. 2002; 30:47.

2. Eerola J, Kananen L, Manninen K, Hellstrom O, Tienari PJ and Hovatta I. No evidence for shorter leukocyte telomere length in Parkinson's disease patients. J Gerontol A Biol Sci Med Sci. 2010; 65:1181-84.

3. Kananen L, Surakka I, Pirkola S, Suvisaari J, Lonnqvist J, Peltonen L, Ripatti S and Hovatta I. Childhood adversities are associated with shorter telomere length at adult age both in individuals with an anxiety disorder and controls. PLoS One. 2010; 5:10826.

4. Kao HT, Cawthon RM, Delisi LE, Bertisch HC, Ji F, Gordon D, Li P, Benedict MM, Greenberg WM and Porton B. Rapid telomere erosion in schizophrenia. Mol Psychiatry. 2008; 13:118-19.

SUPPLEMENTARY TABLES

Supplementary Table 1. Information on genetic variants of LTL used in SATSA

SNP	Chr	Position	Gene	Effect allele	Other allele	Effect allele frequency	Imputed*
**rs11125529**	2	54329370	*ACYP2*	C	A	0.83	Yes
**rs10936599**	3	170974795	*TERC*	T	C	0.25	Yes
**rs7675998**	4	164227270	*NAF1*	A	G	0.19	Yes
**rs2736100**	5	1339516	*TERT*	A	C	0.48	No
**rs9420907**	10	105666455	*OBFC1*	A	C	0.89	Yes
**rs8105767**	19	22007281	*ZNF208*	A	G	0.68	Yes
**rs2281929**	20	61892524	*RTEL1*	T	C	0.89	No

*Imputed SNPs with proper_info>0.4; LTL, leukocyte telomere length

Supplementary Table 2. Crude and adjusted models including interactions with leukocyte telomere length as outcome variable for SATSA cross-sectional

	Model 1[§] estimate (95% CI)	Model 2[£] estimate (95% CI)	Model 3[†] estimate (95% CI)
**SATSA**			
Age (years)	-0.0019 (-0.0032- -0.0006)**	-0.0043 (-0.0092--0.0007)	-0.0022 (-0.0036- -0.0009)**
Sex		-0.0357 (-0.2353-0.1639)	0.0488 (0.0170-0.0807)**
Age*Sex		0.0013 (-0.0016-0.0041)	
GRS			-0.0145 (-0.0942-0.0652)
Age*GRS			0.0001 (-0.0008-0.0010)
Sex*GRS			-0.0010 (-0.0260-0.0240)

**p-value<0.01; [§]Model adjusted for twinness [£]Sex included as a covariate [†]Sex and GRS included as covariates. Reference group for sex is men. GRS, genetic risk score.

Supplementary Table 3. Sex-stratified models of the association between leukocyte telomere length and age for SATSA cross-sectional

	Model 1-Men estimate (95% CI)	Model 2-Women estimate (95% CI)
**SATSA**	N=264	N=372
Age	-0.0030 (-0.0054- -0.0007)*	-0.0018 (-0.0033- -0.0002)*

*p-value<0.05; Models adjusted for twinness.

Supplementary Table 4. SATSA longitudinal characteristics by sex and IPT occasion

In-person testing (IPT)	IPT3	IPT5	IPT6	IPT8	IPT9
Year	1992-1994	1999-2001	2002-2004	2008-2010	2010-2012
Men	(N=162)	(N=149)	(N=126)	(N=122)	(N=102)
Telomere length (mean±sd)[$]	0.66±0.20	0.71±0.11	0.70±0.14	0.70±0.13	0.69±0.15
Age, yrs (mean±sd)	67.42±8.09	68.96±9.43	71.61±9.01	74.39±8.05	76.51±7.53
Women	(N=206)	(N=238)	(N=167)	(N=175)	(N=151)
Telomere length (mean±sd)[$]	0.72±0.20	0.72±0.13	0.72±0.13	0.73±0.11	0.71±0.14
Age, yrs (mean±sd)	69.64±9.51	71.20±10.41	73.01±9.57	76.95±8.82	79.08±8.77

[$]Mean telomere length is adjusted for batch effect and re-scaled back to T/S-ratio.

Supplementary Table 5. Fixed and random effects of the 1 slope model (Model 2) from latent growth curve analysis of leukocyte telomere length and age

<table>
<tr><th colspan="7">Fixed Effects</th></tr>
<tr><th colspan="2">Intercept (estimate (95% CI))</th><th colspan="3">Slope 1 (estimate (95% CI))</th><th colspan="2">Sex (estimate (95% CI))</th></tr>
<tr><td colspan="2">0.7256 (0.7094-0.7418)***</td><td colspan="3">-0.0018 (-0.0028- -0.0009)**</td><td colspan="2">-0.0399 (-0.0626- -0.0173)**</td></tr>
<tr><th colspan="7">Random Effects</th></tr>
<tr><th>$varI_t$</th><th>$varI_p$</th><th>$varS_{1t}$</th><th>$varS_{1p}$</th><th>$\mathit{R}i_ts_{1t}$</th><th>$\mathit{R}i_ps_{1p}$</th><th>Residual</th></tr>
<tr><td>0.0025**</td><td>0.0078***</td><td>0.000005</td><td>0.00002**</td><td>-1.0</td><td>-0.45**</td><td>0.012***</td></tr>
</table>

**p-value<0.01. ***p-value<0.001. Random effects: varI = variance around the intercept; $varS_1$ = variance around slope 1; Ri▯s_1 = correlation between the random intercepts and slope 1; residual = residual variance reflecting within-wave variance. In all models, sex as covariate as well as random effects between twin pairs (subscript 'p') and within twin pairs (subscript 't') were included

Supplementary Table 6. Fixed and random effects of the full two-slope model (Model 3) from latent growth curve analysis of leukocyte telomere length and age including correlation and residual estimates.

<table>
<tr><th colspan="7">Fixed Effects</th></tr>
<tr><th colspan="2">Intercept
(estimate (95% CI))</th><th colspan="2">Slope 1
(estimate (95% CI))</th><th colspan="2">Slope 2
(estimate (95% CI))</th><th>Sex
(estimate (95% CI))</th></tr>
<tr><td colspan="2">0.7287
(0.7095-0.7479)***</td><td colspan="2">-0.0012
(-0.0033-0.0010)</td><td colspan="2">-0.0021
(-0.0034- -0.0008)**</td><td>-0.0409
(-0.0637- -0.0182)***</td></tr>
<tr><th colspan="7">Random Effects</th></tr>
<tr><th>$varI_t$</th><th>$varI_p$</th><th>$varS_{1t}$</th><th>$varS_{1p}$</th><th>$varS_{2t}$</th><th colspan="2">$varS_{2p}$</th></tr>
<tr><td>0.0060***</td><td>0.0082***</td><td>0.00006**</td><td>0.00001</td><td>0.00002*</td><td colspan="2">0.00002</td></tr>
<tr><th>$\mathit{R}i_ts_{1t}$</th><th>$\mathit{R}i_ps_{1p}$</th><th>$\mathit{R}i_ts_{2t}$</th><th>$\mathit{R}i_ps_{2p}$</th><th>$\mathit{R}s_{1t}s_{2t}$</th><th>$\mathit{R}s_{1p}s_{2p}$</th><th>Residual</th></tr>
<tr><td>0.43*</td><td>-0.18</td><td>-1.00</td><td>-0.51**</td><td>1.00</td><td>-0.41</td><td>0.011***</td></tr>
</table>

*p-value<0.05; **p-value<0.01; ***p-value<0.001; Random effects: varI = variance around the intercept; $varS_1$ = variance around slope 1; $varS_2$ = variance around slope 2; Ris = correlation between the random intercept and slope 1 or slope 2; Rs1s2 = correlation between slope 1 and slope 2; residual = residual variance reflecting within-wave variance. In all models, sex as covariate as well as random effects between twin pairs (subscript 'p') and within twin pairs (subscript 't') were included.

Supplementary Table 7. Fixed and random effects of the full spline two-slope model (model 3) from latent growth curve analysis of leukocyte telomere length and age with GRS

<table>
<tr><th colspan="7">Fixed Effects</th></tr>
<tr><th colspan="2">Intercept
(estimate (95% CI))</th><th>Slope 1
(estimate (95% CI))</th><th>Slope 2
(estimate (95% CI))</th><th>Sex
(estimate (95% CI))</th><th colspan="2">GRS
(estimate (95% CI))</th></tr>
<tr><td colspan="2">0.7306
(0.7106-0.7507)***</td><td>-0.0010
(-0.0032-0.0013)</td><td>-0.0023
(-0.0037- -0.0010)**</td><td>-0.0383
(-0.0618- -0.0148)**</td><td colspan="2">-0.0108
(-0.0179- -0.0038)**</td></tr>
<tr><th colspan="7">Random Effects</th></tr>
<tr><th>$varI_t$</th><th>$varI_p$</th><th>$varS_{1t}$</th><th>$varS_{1p}$</th><th>$varS_{2t}$</th><th colspan="2">$varS_{2p}$</th></tr>
<tr><td>0.0053**</td><td>0.0093***</td><td>0.00006*</td><td>0.00002</td><td>0.00002</td><td colspan="2">0.00002</td></tr>
<tr><th>$\mathit{R}i_ts_{1t}$</th><th>$\mathit{R}i_ps_{1p}$</th><th>$\mathit{R}i_ts_{2t}$</th><th>$\mathit{R}i_ps_{2p}$</th><th>$\mathit{R}s_{1t}s_{2t}$</th><th>$\mathit{R}s_{1p}s_{2p}$</th><th>Residual</th></tr>
<tr><td>0.56**</td><td>-0.16</td><td>-1.00</td><td>-0.58**</td><td>0.64</td><td>-0.24</td><td>0.011***</td></tr>
</table>

*p-value<0.05; **p-value<0.01; ***p-value<0.001; GRS, Genetic Risk Score; Random effects: varI = variance around the intercept; varS1 = variance around slope 1; varS2 = variance around slope 2; Ris = correlation between the random intercept and slope 1 or slope 2; Rs1s2 = correlation between slope 1 and slope 2; residual = residual variance reflecting within-wave variance. In all models, sex as covariate as well as random effects between twin pairs (subscript 'p') and within twin pairs (subscript 't') were included.

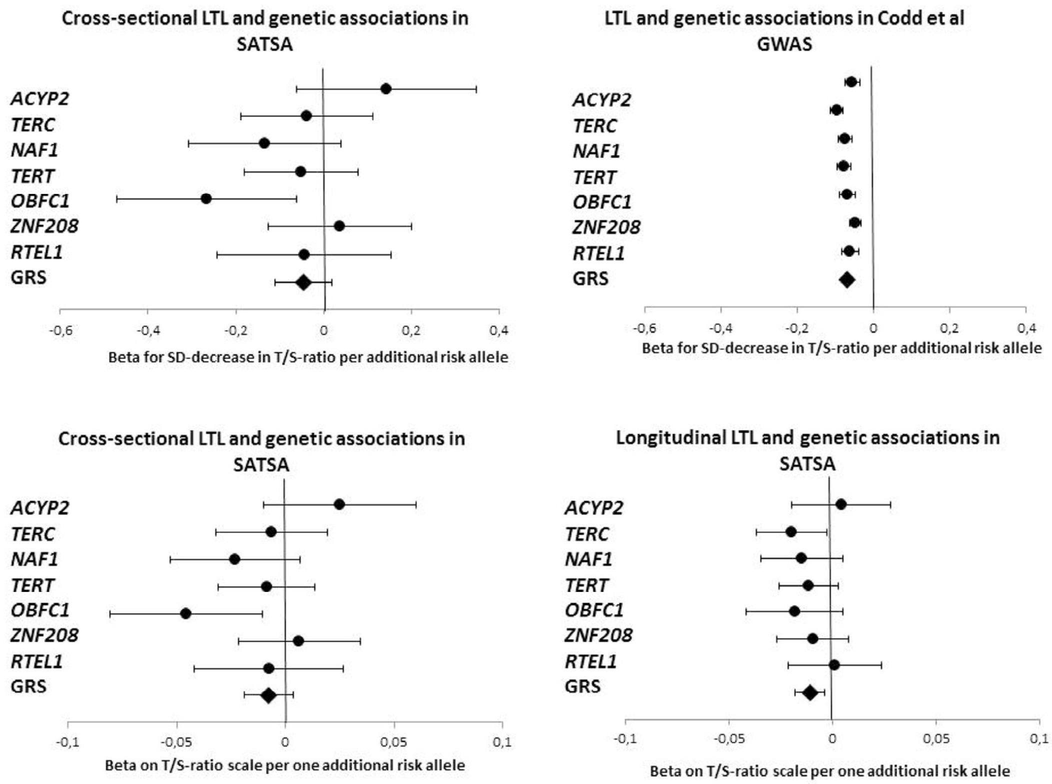

Supplementary Figure 1. Genetic associations between every genetic variant alone and in combination in a genetic risk score (GRS) with leukocyte telomere length (LTL). Left panels are cross-sectional associations in the SATSA study (n=585) with adjustments for family structure, age and sex. Upper panel shows z-transformed LTL values. Lower panel shows LTL values adjusted for batch effect and rescaled back to T/S-ratio scale. Upper right panel presents genetic estimates for LTL from the Codd et al paper (Nature Genet 2013). Lower right panel presents genetic estimates from longitudinal LTL analysis using latent growth curve models.

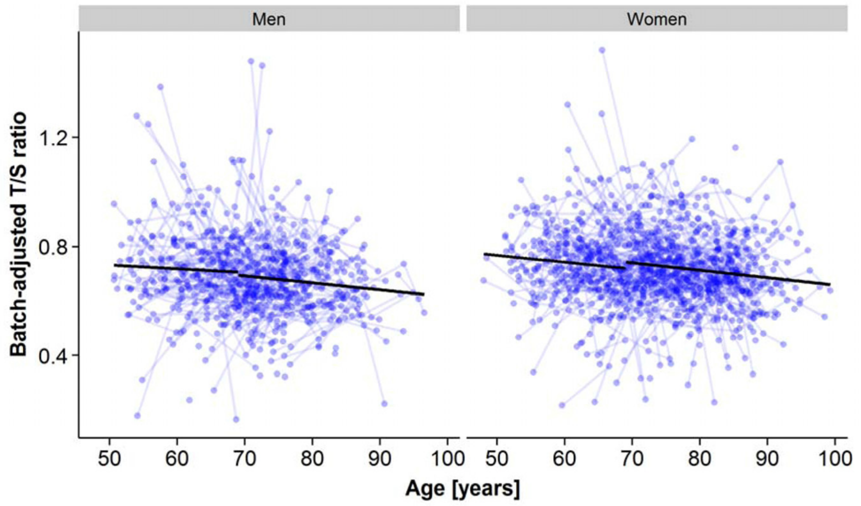

Supplementary Figure 2. Plot of individual trajectories across measurement points longitudinally. The x-axis represents age at each in-person testing (IPT), and the y-axis represents the plate-adjusted residuals of telomere length reported by T/S-ratio. Left panel is men only and right is women only. Each line represents a participant's trajectory from raw data; individuals with only one measurement occasion are presented as single points. In addition, two linear regression lines are shown, before and one after the centering age (69.3 years).

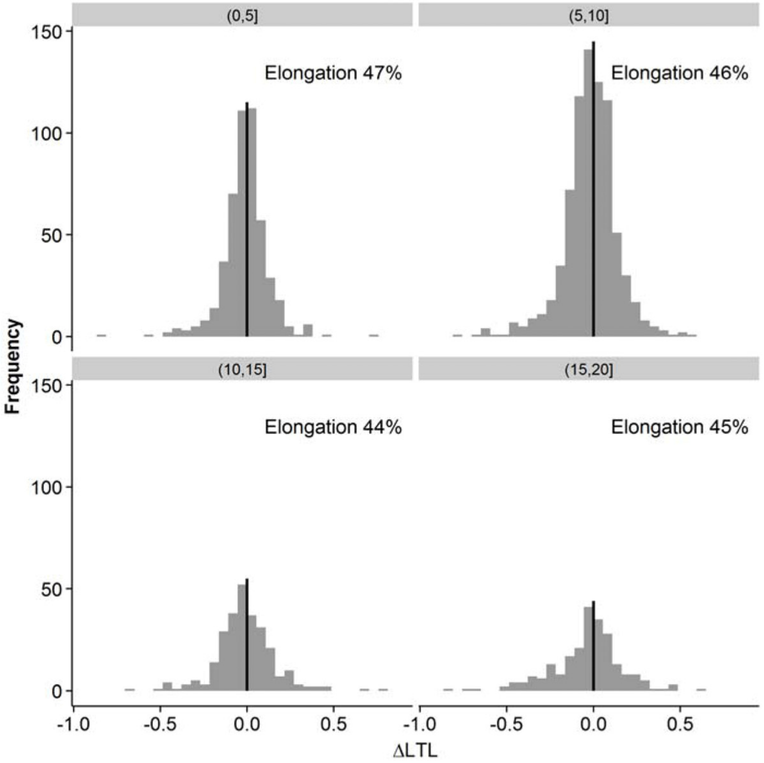

Supplementary Figure 3. Individual relative leukocyte telomere length (LTL) elongation in the longitudinal cohort. The difference in telomere length measurement between two time points is on the x-axis. The frequency is on the y-axis. Telomere elongation is exhibited in samples with delta LTL >0, and telomere attrition is exhibited in samples with delta LTL<0. The four different panels represent the number of years that differs between the two time points of LTL measurements (0->5; 5->10; 10->15; 15->20 years).

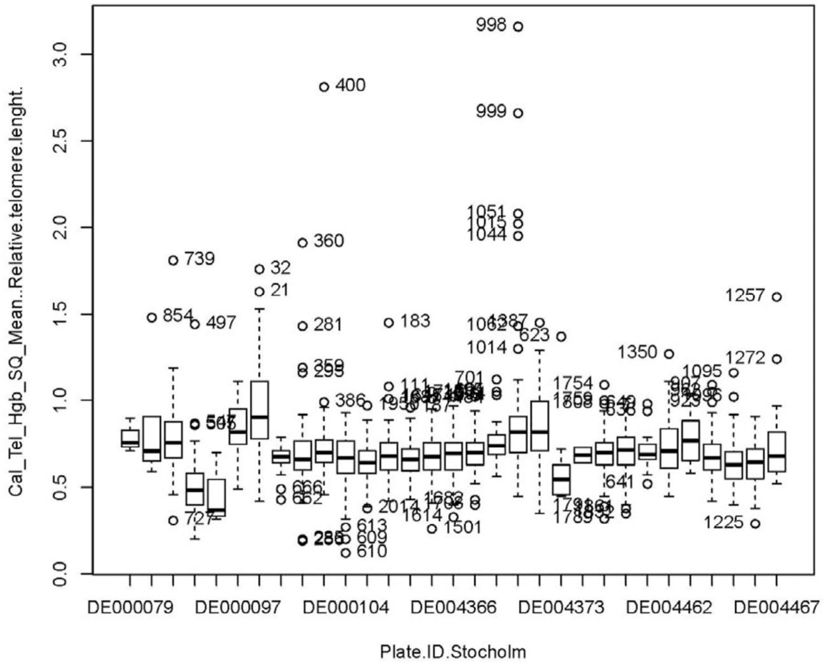

Supplementary Figure 4A. Boxplots demonstrating the measures of telomere length by T/S-ratio over different plates. (**A**) Before adjusting for plate effect.

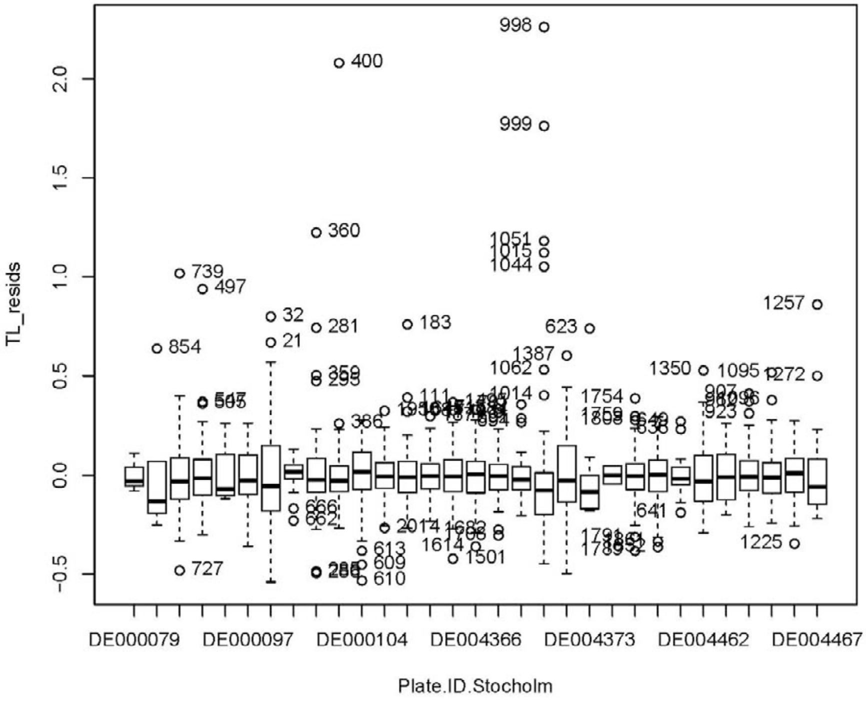

Supplementary Figure 4B. Boxplots demonstrating the measures of telomere length by T/S-ratio over different plates. (**B**) After adjusting for plate effects showing residuals for the association between telomere length and plate ID.

Appendix

SAS code for LGC models used with random effects for within-, and between twinpairs.

```
/*Defining centering age and ages for slopes 1 and 2.*/
data allsubjs;
set allsubjs;
 cage=69.3; /*centering age*/
 iage=round((age-cage),.5); /*centering around cage*/
 Cage1=round((age-cage),.5); if age > 69.3 then Cage1=0; /*Slope 1*/
 Cage2=round((age-cage),.5); if age < 69.3 and age ne . then Cage2=0; /*Slope
2*/
run;

/*Intercept model.*/
title1 'Random Effects Model Fitting';
title2 'centering=69.13: selected=0';
title3 'Regular model: Intercept with sex';
proc mixed data=allsubjs noitprint covtest IC noclprint method=ML;
           class pairid twinid sex;
           model tlresidual=sex/solution ddfm=bw CL;
           random intercept/sub=twinid(pairid) type=unr;
           random intercept/SUB=pairid type=unr;
run;

/*One-slope model.*/
title3 'Regular model: L&S with sex';
proc mixed abs scoring=5 maxfunc=300 data=allsubjs itdetails covtest IC
noclprint method=ML;
           class pairid twinid sex;
           model tlresidual=iage sex/solution ddfm=bw outp=bpred CL;
           random intercept iage/SUB=twinid(pairid)type=unr;
           random intercept iage/SUB=pairid TYPE=unr;
run;

/*Two-slope model.*/
title3 'Two-Slope Model with sex';
proc mixed abs data=allsubjs scoring=300 maxfunc=300 itdetails covtest IC
noclprint method=ML;
           class pairid twinid sex;
           model tlresidual=Cage1 Cage2 sex/solution ddfm=bw  outp=bpred CL;
           random intercept Cage1 Cage2/sub=twinid(pairid) type=unr;
           random intercept Cage1 Cage2/sub=pairid TYPE=unr;
run;
```
